# Does previous stroke modify the relationship between inflammatory biomarkers and clinical endpoints in CKD patients?

**DOI:** 10.1186/s12882-021-02625-2

**Published:** 2022-01-18

**Authors:** James Tollitt, Stuart M. Allan, Rajkumar Chinnadurai, Aghogho Odudu, Margaret Hoadley, Craig Smith, Philip A. Kalra

**Affiliations:** 1 Renal Department, Salford Royal NHS Trust, Stott Lane, Salford, M6 8HD UK; 2 Division of Cardiovascular Sciences, University of Manchester, Oxford Road, Manchester, UK; 3 Division of Neuroscience and Experimental Psychology, School of Biological Sciences, Faculty of Biology, Medicine and Health, University of Manchester, Manchester, M13 9PT UK; 4 Geoffrey Jefferson Brain Research Centre, The Manchester Academic Health Science Centre, Northern Care Alliance NHS Group, University of Manchester, Manchester, UK; 5 Renal Department, Manchester Foundation Trust, Manchester, UK; 6 Stroke Department, Salford Royal NHS Trust, Salford, UK

**Keywords:** Stroke, CKD, Inflammation, Biomarkers, Mortality

## Abstract

**Background:**

Chronic kidney disease (CKD) is an independent risk factor for stroke. Stroke is also an independent risk factor for worse CKD outcomes and inflammation may contribute to this bidirectional relationship. This study aims to investigate inflammatory biomarkers in patients with non-dialysis CKD (ND-CKD) with and without stroke.

**Methods:**

A propensity matched sample from > 3000 Salford Kidney Study (SKS) patients, differentiated by previous stroke at study recruitment, had stored plasma analyzed for interleukin- 6 (IL-6), Von Willebrand Factor (VWF) and C-reactive protein (CRP). Multivariable cox regression analysis investigated associations between inflammation and death, end-stage renal disease (ESRD) and future non-fatal cardiovascular events (NFCVE).

**Results:**

A total of 157 previous stroke patients were compared against 162 non-stroke patients. There were no significant differences in inflammatory biomarkers between the two groups. Previous stroke was associated with greater mortality risk, hazard ratio (HR) (95% CI) was 1.45 (1.07–1.97). Higher inflammatory biomarker concentrations were independently associated with death but not ESRD or NFCVE in the total population. For each 1 standard deviation (SD) increase in log IL-6, VWF and CRP, the HR for all-cause mortality were 1.35 (1.10–1.70), 1.26 (1.05–1.51) and 1.34 (1.12–1.61), respectively. CRP retained its independent association (HR 1.47 (1.15–1.87)) with death in the stroke population.

**Conclusion:**

Previous stroke is an important determinant of mortality. However, the adverse combination of stroke and ND-CKD does not seem to be driven by higher levels of inflammation detected after the stroke event. Biomarkers of inflammation were associated with worse outcome in both stroke and non-stroke ND-CKD patients.

**Trial registration:**

15/NW/0818.

**Supplementary Information:**

The online version contains supplementary material available at 10.1186/s12882-021-02625-2.

## Introduction

Chronic Kidney Disease (CKD) is a risk factor for stroke, more severe stroke and recurrent stroke [1–5]. The increased risk is not fully explained by the aggregation of traditional risk factors within the CKD population [3]. This interaction is further complicated because previous stroke is an independent risk factor for incident CKD, reaching end-stage renal disease (ESRD), future cardiovascular events and all-cause mortality [6–8]. This bi-directional association of worse outcomes for both conditions requires further investigation to understand possible mechanisms.

Systemic inflammation is thought to be a contributor to worse outcomes for patients with CKD [9, 10] and more rapid CKD progression [11]. Inflammatory biomarkers in acute stroke are also able to predict stroke outcomes [12] and could provide future therapeutic targets [13–15]. There is also emerging evidence of the usefulness of inflammatory biomarkers to predict risk of stroke [16] and other cardiovascular disease [17]. However, the role of inflammation in predicting a stroke in patients with CKD and the prognostic role of inflammation in patients with both previous stroke and non-dialysis CKD (ND-CKD) have not previously been investigated.

This study hypothesised that vascular and systemic inflammation would be higher in stroke patients with ND-CKD than a propensity matched cohort and that higher levels of inflammation would predict poor long-term outcomes in stroke and non-stroke ND-CKD populations.

## Materials and methods

The Salford Kidney Study (SKS) is a longitudinal epidemiological cohort study of more than 3000 adults with all-cause ND-CKD recruited since October 2002 [18, 19]. Ethical approval was granted by the North West - Greater Manchester South Research Ethics Committee (REC15/NW/0818). All methods were performed in accordance with the principals set out by the Declaration of Helsinki. Inclusion criteria were patients 18 years or older, referred to a tertiary renal centre, an eGFR < 60 mL/min/1.73m^2^ and not requiring immediate renal replacement therapy. Demographic, comorbidity and laboratory data were recorded at baseline and annually. Study blood samples were taken at baseline and at annual review, immediately centrifuged and plasma stored at -80^0^c. Self-reported cerebrovascular and cardiovascular events and event dates were validated following review of clinical records, radiology reports, general practice records, clinical coding and outpatient clinic letters. A previous validation exercise suggested < 1% variance between the GP records and secondary care electronic health records.

**Fig. 1:**
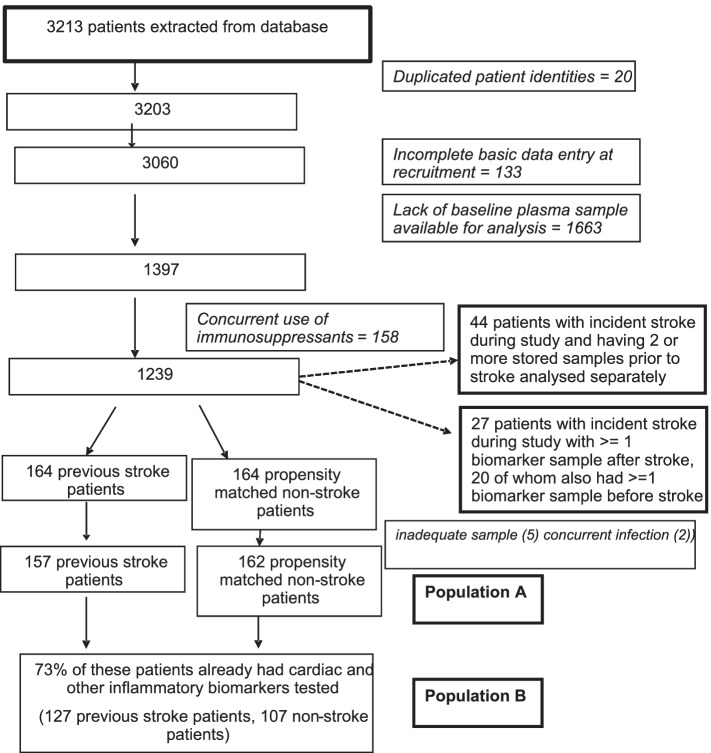
Consort diagram for patient population

This project consisted of two sub studies examining two overlapping patient populations, population A and population B. Population A was created by searching the SKS database for patients who had a stroke at study recruitment and had stored plasma available for analysis. This group of patients were propensity matched 1:1 against other patients in the SKS who had no historical stroke. This created population A. Population A had plasma inflammatory biomarkers analysed (interleukin- 6 (IL-6), Von Willebrand Factor (VWF) and C-reactive protein (CRP)),

Population B comprised of patients who were characterised in population A but had further stored inflammatory and cardiac biomarker results available in the SKS biobank. These patients must have had biomarkers taken from plasma samples taken at the same time point as the new inflammatory biomarkers which we were studying. Population B therefore represents a a well-matched and large subgroup of population A. No patients were included in population B who were not included in population A. The inflammatory markers stored and available in the SKS biobank were: Neutrophil gelatinase-associated lipocalin (NGAL), auto-antibodies to Anti-Apolipoprotein A-1 (Anti-Apo A1 IgG) and myeloperoxidase (MPO) and cardiac biomarkers (high sensitive cardiac troponin T (HS-CTnT) and N-terminal pro brain natriuretic peptide (NT- proBNP)). Consort diagram is summarised in Fig. 1. Cardiac biomarkers were included in the results to demonstrate that the propensity matching had appropriately matched non-stroke study participants who had a similar cardiac risk profile [20]. Imputation of missing data would not be appropriate as data was not missing at random.

A composite outcome of non-fatal cardiovascular events (NFCVE) comprised myocardial infarction, coronary revascularization (including bypass grafting or angioplasty), cardiac arrest, cerebrovascular events, and newly diagnosed peripheral vascular disease including amputation. Patients were followed from study recruitment until death, commencement of renal replacement therapy or eGFR < 10 mL/min/1.73m^2^ (using modified diet in renal disease formula) [21]. For patients not reaching study end points, data were censored on the last hospital visit or on 2nd March 2018.

### Statistical analysis

Analyses were performed using SPSS version 23.0. Propensity matching (matched for age, ethnic group, sex, baseline eGFR, baseline proteinuria, diabetes, atrial fibrillation, hypertension, hypercholesterolemia, smoking history, previous myocardial infarction and statin use) was generated on a 1:1 basis using nearest neighbour methodology. Propensity matching was performed using “MatchIt” package of R software [22, 23]. Univariate analyses were performed stratified by the presence or absence of previous stroke at study entry. Between group comparisons were made using Kolmogorov Simonov (non-parametric) or t-Test (parametric) tests for continuous variables and the chi square test for categorical variables. Unadjusted survival was assessed using Kaplan-Meier methodology and significance was assessed using the log rank test. Adjusted survival analyses were performed using multivariable cox regression for the mortality end point in population A. Variables included in the models were selected a priori on the basis of factors known in the literature to be associated with poor CKD outcomes, even if they did not have a significant association with a clinical outcome in univariate analysis. In order to account for competing risks, the HR were derived by censoring at the competing event [24]. All HR are presented alongside 95% confidence intervals (CI). A *p* value of ≤0.05 was considered statistically significant for all analyses.

The inflammatory biomarker results were analysed as continuous variables with a HR reported per standard deviation (SD) increase in biomarker log transformed concentration. Several samples had results below the minimal level for detection. We arbitrarily assigned a value of 0.0001 for these samples.

## Results

There were 157 patients with a previous stroke before study entry: 105 (66.9%) were ischemic, 11 hemorrhagic (7.0%), 3 had 2 strokes of differing etiologies (1.9%) and 38 strokes (24.2%) were not definitively classified, due to the unavailability of radiology reports from the time of stroke. Time between the previous stroke event and blood draw for inflammatory biomarker analysis in population A was a median of 88 (IQR 41–172) months. Time between previous stroke event and blood draw for inflammatory and cardiac biomarker blood draw for population B was 87 (IQR 160–41) months. The propensity matched population (population A) consisted of 162 patients without a stroke before study entry. Patients were well matched between the groups although more patients with a diagnosis of renovascular disease (48.4% v 35.2% *p* = 0.02) were included in the previous stroke group and less patients with a previous stroke had ND-CKD as a result of “unknown” cause (14.0% v 20.2% *p* < 0.01) (Table 1). Biochemical and hematological comparisons at baseline are found in Table S1a. 234 (73.3%) of the patients also had additional inflammatory and cardiac biomarkers performed at the same baseline date (population B). Population B represented an unselected subgroup of population A who had stored inflammatory biomarker results in our biobank. We opportunistically analyzed population B to help answer our hypothesis. Baseline characteristics of this population are demonstrated in Table 2 and biochemical and hematological comparisons are found in Table S1b.

**Table 1:** Baseline characteristics for population A between the previous stroke group and propensity matched sample

		Previous stroke		
		No *N* = 162	Yes *N* = 157	*p*-value
Age (years)		73 (67–79)	73 (67–78)	0.83
Male Gender		113 (69.8%)	113 (72.0%)	0.70
Living alone		33 (20.4%)	33 (21.0%)	0.91
Widowed		31 (19.1%)	28 (17.6%)	0.57
Ethnic group	Caucasian	157 (96.9%)	153 (97.5%)	0.11
Aetiology of Renal Disease	Renovascular Disease/Hypertension	57 (35.2%)	76 (48.4%)	0.02*
	Diabetic kidney disease	32 (19.8%)	33 (21.0%)	0.78
	Glomerulonephritis/ Vasculitis	12 (7.4%)	13 (8.3%)	0.77
	Pyelonephritis	4 (2.5%)	5 (3.2%)	0.70
	Autosomal Dominant Polycystic Kidney Disease	8 (4.9%)	8 (5.1%)	0.95
	Other/Unknown	49 (30.2%)	22 (14.0%)	< 0.01*
Smoking history		108 (66.7%)	112 (76.7%)	0.10
Diabetes		76 (46.9%)	71 (45.2%)	0.76
Hypertension		161 (99.4%)	157 (98.7%)	0.55
Myocardial infarction (MI)		43 (26.5%)	43 (27.0%)	0.93
Coronary Artery Disease (CAD)		82 (50.6%)	73 (46.5%)	0.54
Heart failure (HF)		56 (34.6%)	50 (31.8%)	0.61
Peripheral vascular disease (PVD)		48 (29.6%)	45 (28.7%)	0.85
Any MI, HF, CAD or PVD		116 (71.6%)	100 (63.7%)	0.13
Atrial fibrillation		14 (8.6%)	23 (14.6%)	0.09
Current malignancy		2 (1.2%)	3 (1.9%)	0.46
	Statin	125 (77.2%)	124 (77.9%)	0.86
	RAS blockade	100 (61.7%)	100 (62.9%)	0.83
Blood Pressure at study recruitment	Systolic	145 (132–159)	142 (128–155)	0.31
	Diastolic	71 (64–80)	70 (62–80)	0.68
Albumin (g/l)		43 (40–44)	42 (40–44)	0.28
Corrected calcium (mmol/L)		2.32 (2.18–2.41)	2.29 (2.20–2.37)	0.10
Phosphate (mmol/l)		1.14 (0.99–1.29)	1.12 (0.98–1.32)	0.97
Haemoglobin (g/L)		118 (107.5–128.0)	120 (109.0–132.0)	0.52
eGFR (mL/min/1.73m^2^)		22.0 (14.8–30.0)	23.0 (15.0–33.5)	0.36

Continuous variables expressed as median (interquartile range) and categorical variables presented as number (%). eGFR calculated using CKD-MDRD formula. Coronary artery disease includes myocardial infarction, coronary artery bypass grafting or angioplasty and cardiac arrest. Blood pressure taken using automated sphygmomanometer with an appropriately sized cuff after at least 5 min of seated rest. *Abbreviations:* *RAS* *blockade*, Renin angiotensin system blockade. *eGFR *estimated glomerular filtration rate (MDRD)

**Table 2:** Baseline characteristics of 73% sample (population B) of patients who had previously undergone cardiac and other inflammatory biomarker analyses

		Previous stroke		
		No *N* = 107	Yes *N* = 127	*p*-value
Age (years)		72 (66–77)	72 (66–78)	0.76
Male Gender		75 (70.1%)	91 (71.7%)	0.79
Living alone		17 (15.9%)	24 (18.9%)	0.55
Widowed		18 (16.8%)	22(17.3%)	0.92
Ethnic group	Caucasian	104 (97.2%)	124 (97.6%)	0.33
Aetiology of renal disease	Renovascular Disease/Hypertension	48 (44.9%)	65 (51.2%)	0.34
	Diabetic kidney disease	22 (20.6%)	28 (22.0%)	0.78
	Glomerulonephritis/ Vasculitis	8 (7.5%)	10 (7.9%)	0.91
	Pyelonephritis	3 (2.8%)	4 (3.1%)	0.88
	Autosomal Dominant Polycystic Kidney Disease	6 (5.6%)	6 (4.7%)	0.76
	Other/Unknown	20 (18.7%)	14 (11.0%)	0.10
Smoking history		74 (69.8%)	88 (75.9%)	0.31
Diabetes		50 (46.7%)	57 (44.9%)	0.78
Hypertension		107 (100%)	126 (99.2%)	0.36
Myocardial infarction		31 (29.0%)	33 (26.0%)	0.61
Coronary artery disease		58 (54.2%)	60 (47.2%)	0.29
Heart failure		40 (37.4%)	40 (31.5%)	0.34
Peripheral vascular disease		38 (35.5%)	39 (30.7%)	0.44
Any MI, HF, CAD or PVD		82 (76.6%)	81 (63.8%)	0.03*
Atrial fibrillation		8 (7.5%)	18 (14.2%)	0.10
Current malignancy		1 (0.9%)	3 (2.4%0	0.40
	Statin	87 (81.3%)	99 (78.0%)	0.53
	RAS blockade	69 (64.5%)	80 (63.0%)	0.81
Albumin (g/l)		43 (41–45)	42 (40–45)	0.09
Corrected calcium (mmol/L)		2.23 (2.16–2.36)	2.25 (2.18–2.34)	0.71
Phosphate (mmol/l)		1.13 (1.00–1.28)	1.13 (0.98–1.34)	0.75
Haemoglobin (g/L)		119.0 (109.0–128.0)	121.0 (112.0–132.0)	0.21
eGFR (mL/min/1.73m^2^)		21.4 (14.9–29.5)	23.1 (15.4–35.5)	0.16

Continuous variables expressed as median (interquartile range) and categorical variables presented as number (%). eGFR calculated using CKD-MDRD formula. Coronary artery disease includes myocardial infarction, coronary artery bypass grafting or angioplasty and cardiac arrest. *Abbreviations:* *RAS blockade *Renin angiotensin system blockade. *eGFR* estimated glomerular filtration rate (MDRD)

### Biomarkers at study recruitment

There were no significant differences in concentrations of plasma IL-6, CRP and VWF between the propensity matched groups in population A (Table 3). In study population B, higher levels of MPO and Anti-Apo A1 were noted in patients with a previous stroke. Similar findings were demonstrated when confining the propensity matched comparison to the patients with definitive ischemic strokes (*N* = 105) (*p* value for significant difference in Anti-Apo A1 IgG was 0.05 and for MPO was < 0.01).

**Table 3:** The differences in baseline inflammatory, cardiac and kidney biomarker values in population A and population B

		Stroke at recruitment		*p*-value
		**Population A**		
Type of Biomarker		No N = 162	Yes N = 157	
Inflammatory	Interleukin 6 (pg/mL)	4.70 (2.77–7.51)	4.65 (3.00–7.60)	0.31
	Von Willebrand Factor (IU/mL)	2.1 (1.5–2.7)	2.0 (1.6–2.7)	0.45
	C-reactive protein (mg/L)	2.8 (1.6–5.8)	3.6 (1.8–9.2)	0.10
Kidney Injury	eGFR (mL/min/1.73m^2^)	22.0 (14.8–30.0)	23.0 (15.0–33.5)	0.36
		**Population B**		
		N = 107	N = 127	
Inflammatory	Interleukin 6 (pg/mL)	4.35 (2.75–6.59)	4.89 (3.18–7.60)	0.22
	Von Willebrand Factor (IU/mL)	2.0 (1.4–2.6)	2.0 (1.6–2.7)	0.37
	C-reactive protein (mg/L)	2.7 (1.6–6.0)	3.6 (1.8–9.2)	0.14
	NGAL (ng/mL)	283 (200–430)	263 (190–397)	0.26
	MPO (ng/mL)	33.0 (17.6–57.3)	44.1 (26.1–69.5)	0.04*
Cardiac	Anti-Apo A1 (AU)	0.48 (0.35–0.65)	0.58 (0.33–0.89)	0.04*
	NT proBNP (pg/mL)	625 (238–1246)	516 (199–1548)	0.34
	Hs-cTnT (ng/L)	23.6 (13.4–39.5)	24.320 (13.8–36.2)	0.62
Kidney Injury	eGFR (mL/min/1.73m^2^)	21.4 (14.9–29.5)	23.1 (15.4–35.5)	0.16

Data presented using medians and interquartile range. VWF and anti apoA 1 were normally distributed. Only 234 (107 non-stroke, 127 stroke) patients had cardiac, NGAL, MPO biomarkers performed

### Effect of stroke on patient outcomes

**Fig. 2:**
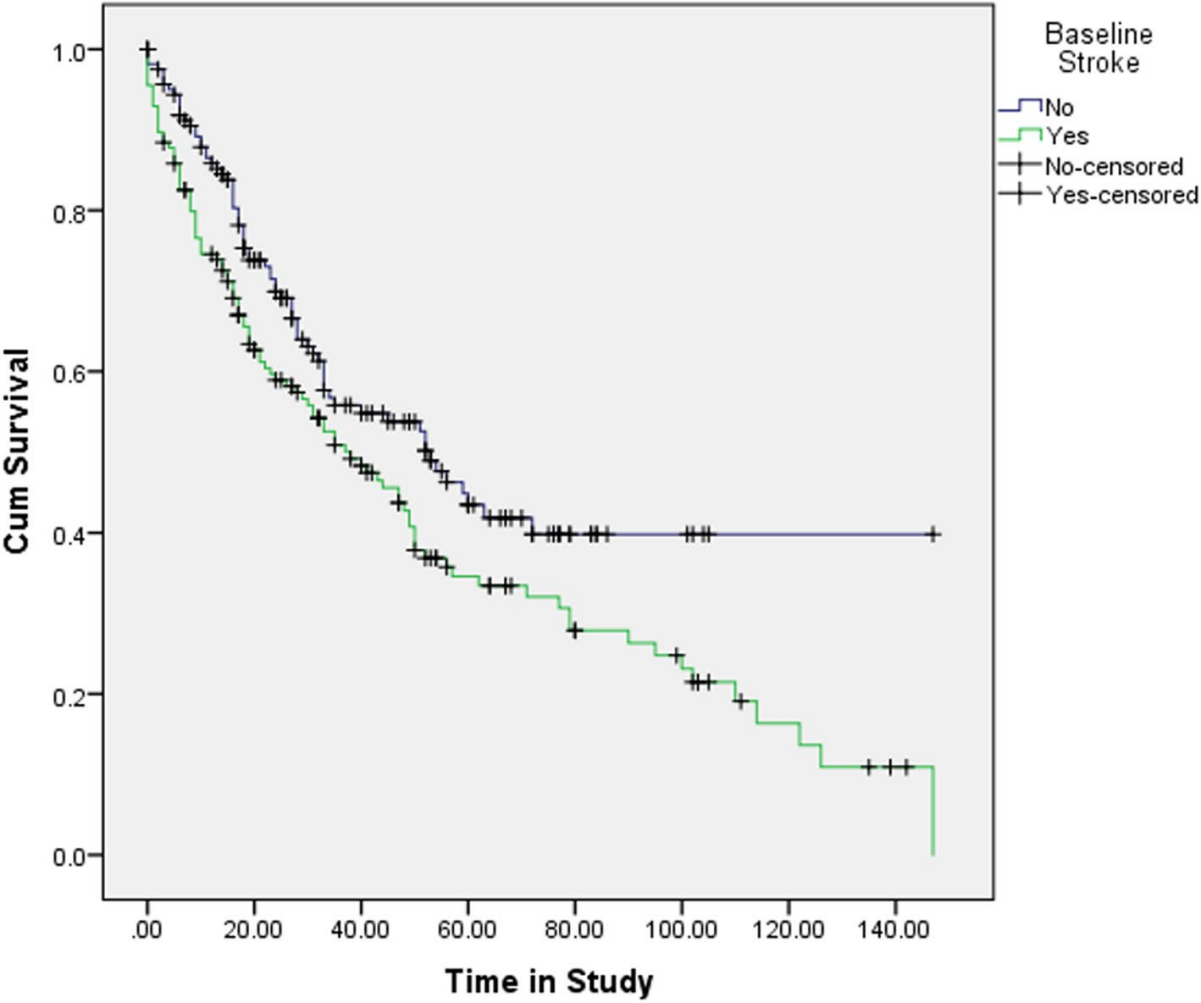
Kaplan Meier curve for all-cause mortality censored at study follow up for patients with a previous stroke at study recruitment compared with patients without (Population A)

Despite a balanced comorbidity burden between groups in population A, the patients with a stroke had significantly worse outcomes. The Kaplan Meier demonstrates a significant difference in unadjusted all-cause mortality (Fig. 2). Median survival in those patients with a previous stroke was 38 months (95%CI 26–49) compared to 53 months (95%CI 32–73) in those without. In cox regression analysis previous stroke demonstrated a HR of 1.47 (95%CI 1.15–1.87). More patients with a previous stroke either had further non-fatal cardiovascular events and/or died. A similar number of patients reached ESRD, but proportionally more patients with a previous stroke received a transplant as their first method of renal replacement therapy 8 (13.1%) v 2 (3.6%) although absolute numbers were small (Table 4).

**Table 4:** Outcomes of Population A

Outcome	Non- Stroke population	Stroke population	P value
eGFR progression slope (mL/min/1.73m^2^/year)^a^	−1.19 (−3.80–1.10)	−1.67 (−3.80–0.08)	0.12
Non-fatal cardiovascular events	13 (8.0%)	42 (26.9%)	< 0.01*
ESRD	56 (34.6%)	61 (38.9%)	0.43
First method of RRT: Dialysis	40 (71.4%)	31 (50.8%)	
Transplant	2 (3.6%)	8 (13.1%)	
eGFR < 10	14 (25.0%)	22 (36.1%)	0.04*
All-cause mortality	72 (44.4%)	103 (65.6%)	< 0.01*
Death before dialysis	44 (61.1%)	58 (56.3%)	0.53
Age at death (years)	79 (74–84)	77 (72–84)	0.27
Months in study^b^	27 (15–52)	24 (9–50)	0.44

a = delta eGFR (MDRD) determined based on linear regression of patients who had more than 2 creatinine measurements during study.300 patients met this criteria (148 no previous stroke, 152 with previous stroke), b = censored for death, ESRD, March 2018, last hospital visit. *Abbreviations:* *eGFR* estimated glomerular filtration rate, *ESRD* End stage renal disease, *RRT* Renal replacement therapy

### Biomarkers and their association with patient outcomes

Multivariable associations between the inflammatory biomarkers are shown in Table 5. Higher levels of inflammation were independently associated with all-cause mortality within the total population. The HR and 95% confidence intervals for each 1SD increase in IL-6, VWF and CRP for all-cause mortality were 1.35 (1.10–1.69), 1.26 (1.05–1.51) and 1.34 (1.12–1.61), respectively. When separated by history of previous stroke, systemic and vascular inflammation (assessed by IL-6 and VWF respectively) no longer significantly contributed to the risk of death. CRP retained its statistical significance after full adjustment for all comorbidities and demographics, irrespective of stroke at baseline (HR 1.47 (1.15–1.87)).

**Table 5:** Multivariable cox regression for all-cause mortality

Total Population						
	IL-6 (per SD)		VWF (per SD)		CRP (per SD)	
	HR (95%CI)	P Value	HR (95%CI)	P Value	HR (95%CI)	P Value
Unadjusted	1.53 (1.28–1.83)	< 0.01*	1.36 (1.15–1.62(	< 0.01*	1.39 (1.18–1.63)	< 0.01*
Model 1	1.42 (1.19–1.70)	< 0.01*	1.28 (1.08–1.52)	< 0.01*	1.35 (1.15–1.59)	< 0.01*
Model 2	1.350 (1.1–1.69)	0.01*	1.26 (1.05–1.51)	0.01*	1.341 (1.12–1.61)	< 0.01*
**No previous stroke population**						
Unadjusted	1.61 (1.24–2.12)	< 0.01*	1.57 (1.21–2.03)	< 0.01*	1.373 (1.08–1.75)	0.01*
Model 1	1.60 (1.20–2.14)	< 0.01*	1.39 (1.06–1.81)	0.02*	1.322 (1.03–1.69)	0.03*
Model 2	1.50 (1.09–2.06)	< 0.01*	1.34 (1.01–1.79)	0.04*	1.23 (0.91–1.66)	0.17
**Previous stroke population**						
Unadjusted	1.46 (1.15–1.85)	< 0.01*	1.18 (0.93–1.49)	0.18	1.390 (1.12–1.72)	< 0.01*
Model 1	1.33 (1.02–1.62)	0.04*	1.17 (0.94–1.47)	0.16	1.36 (1.09–1.69)	0.01*
Model 2	1.20 (0.89–1.62)	0.23	1.19 (0.93–1.52)	0.17	1.47 (1.15–1.87)	< 0.01*

Model 1 adjusted for age and eGFR. Model 2 adjusted for model 1 + myocardial infarction, peripheral vascular disease, diabetes, heart failure, male gender, *uPCR* smoking history, living alone, albumin at baseline

In univariate cox regression analysis, age, male sex, smoking, heart failure, previous myocardial infarction, peripheral vascular disease, higher proteinuria, lower eGFR, lower serum albumin and 1SD increase in all inflammatory biomarkers were associated with all-cause mortality. Male sex, lower eGFR at baseline, higher proteinuria and lower serum albumin were associated with reaching ESRD. Age, previous myocardial infarction, peripheral vascular disease, higher proteinuria and lower albumin were associated with future NFCVE. The univariate associations for the total population and for the population separated by previous stroke are shown in Table S2. Living alone was not associated with all-cause mortality in the total population; however it did demonstrate association with all-cause mortality in patients without a previous stroke (HR 2.55 (95%CI 1.42–4.59)).

## Discussion

This propensity matched study demonstrated that stroke is an independent risk factor for all-cause mortality. The results of this study support the body of evidence, that increased mortality in patients with CKD and stroke is not simply due to an aggregation of traditional cardiovascular risk factors. There must be unmeasured confounders or alternative pathophysiological mechanisms which remain undiscovered or uninvestigated.

Increased levels of systemic and vascular inflammation were independently associated with all-cause mortality in our propensity matched population. This is consistent with the literature that has examined this in haemodialysis patients and in the general population [9, 10, 25–28]. The key finding from this study is that whilst CRP retained significant independent association with mortality in the subgroup of patients with previous stroke, IL-6 and VWF did not. One interpretation of this could be that CRP is a more sensitive and robust measure of peripheral inflammation when studied over a long period of time. A recent systematic review analyzed the role of vascular (e.g. VWF, homocysteine) and systemic (e.g. CRP, IL-6) inflammatory biomarkers in relation to cerebral small vessel disease (SVD) in a non-CKD population. Markers of vascular inflammation, especially amongst patients who had suffered a previous stroke, were associated with SVD in cross sectional analysis. Higher levels of systemic inflammatory mediators were associated with SVD in longitudinal but not cross sectional analyses [29]. In our group of patients with a previous stroke matched with patients with a similar high cardiovascular burden and high levels of inflammation the independent role of vascular and systemic inflammation demonstrates a lower relative contribution to the poor outcomes seen in this population. A recent study correlated numerous inflammatory biomarkers with eGFR in patients after a stroke or TIA. After adjustment for age, the relationship between eGFR and biomarker was at most modest suggesting that putative risk factors such as inflammation are unlikely to be mechanistically important in pathophysiology of stroke in CKD [30]. One proposes that there are other competing pathophysiological mechanisms such as vascular calcification and platelet dysfunction which are more pertinent.

In population B, MPO and Anti Apo A1 were significantly higher in patients who had suffered a stroke compared to those without prior stroke. MPO has been shown to correlate with infarct size, stroke severity and mortality and can be detected up to 3 weeks post infarct [31–33]. MPO is associated with cardiovascular events within the general population yet has been shown to decline with advancing CKD [34–36]. It has been speculated that this inverse correlation between kidney function and MPO is due to uraemic toxins inhibiting MPO enzymatic activity [36]. Conversely in animal models, MPO knockout mice demonstrate amelioration of CKD progression [37]. Other studies have demonstrated that whilst MPO activity reduces with CKD, its oxidation products actually increase with more advanced CKD [38]. In patients with CKD a large US study has shown MPO to be independently associated with CKD progression but not with cardiovascular disease or death [39]. This finding was more pronounced in better preserved kidney function (eGFR > 45 mL/min). The interaction between MPO, the oxidation products of MPOCKD and cardiovascular events requires further interrogation. MPO could prove a useful biomarker in relation to CKD and stroke risk and in relation to outcomes of stroke in patients with CKD. This requires further consideration given the findings in our study.

Antibodies against apolipoprotein A-1 are biomarkers of atherogenesis and are independently associated with cardiovascular events and mortality in the general population [40–42]. There is a dose effect relationship between anti-apo-A1 levels and mortality risk in the general population as well as certain single nucleotide polymorphisms (SNP) in genome wide association studies. These findings suggest there are pre-clinical genetic risk factors associated with mortality and autoimmunity [41]. Future work could consider the presence of pathogenic SNPs in patients with a previous stroke and could interrogate larger databases to understand if antibodies against apolipoprotein A-1 are a significant independent biomarker of cardiovascular disease and mortality in patients with CKD.

This study is unique, it has demonstrated that inflammatory biomarkers may not be the panacea of biomarker research in patients with advanced CKD and an established and significant cardiovascular burden. When considering CKD in broad terms, inflammation is undoubtedly an important contributor to poor outcomes [9, 11, 43, 44]. Some studies which have demonstrated this finding do not adjust for significant comorbidity in their final regression models [11, 43]. When considering a group of patients with significant cardiovascular comorbidity and including most comorbidities within the regression models, inflammation may be less important. Searching for important inflammatory biomarkers in patients with well-established comorbidity risks factors for poor outcomes should be carefully considered. Furthermore, there is an urgent research need to understand what the therapeutic targets for patients with a high cardiovascular risk are so the predictable poor outcomes can be addressed. In the meantime quality improvement methods should be used to ensure that patients with CKD at highest cardiovascular risk are intensively and meticulously optimized in relation to the known best medical therapy and lifestyle advice and interventions [45–47].

Limitations of this study include uncertain generalizability - this propensity matched, high cardiovascular comorbidity population was from a single center, with a predominantly Caucasian population, and there was no data available to adjust or match for socioeconomic status. By the very nature of this study there will be an unquantifiable bias towards stroke survivors. Secondly, despite the propensity matching there may have been unmeasured confounders which differentiated the stroke and non-stroke population. Thirdly, the blood draws for analysis of inflammatory biomarkers occurred many months after the stroke events in most cases, and hence the results cannot be considered definitive. Finally, patients did not undergo brain imaging at the time of blood draw so we cannot be certain that some patients who had suffered silent brain infarction are not included within the non-stroke group.

In conclusion higher levels of inflammation were independently associated with all-cause mortality but not ESRD or future NFCVE in ND-CKD patients with a high cardiovascular comorbidity burden. Previous stroke represents an important independent determinant of worse outcomes in CKD. However, we provide evidence that biomarkers of inflammation on blood drawn months after the stroke event are not higher in patients with CKD and stroke compared with CKD without stroke. This proposes that whilst inflammation is a factor in stroke etiology in patients with and without CKD, it is not the dominant driver of the worse outcomes seen in the CKD and stroke population.

## Supplementary Information

**Additional file 1.**

## Data Availability

All data generated or analyzed during this study are included in this published article.
