# Supplementary material for: Does previous stroke modify the relationship between inflammatory biomarkers and clinical endpoints in CKD patients?
Source: BMC Nephrol. 2022 Jan 18;23:38. doi: 10.1186/s12882-021-02625-2 (PMC8767689; doi:10.1186/s12882-021-02625-2)
Supplement: Supplementary file 1 — Additional file 1. [file 12882_2021_2625_MOESM1_ESM.docx]

## Supplementary Material:

Detailed Laboratory Methods

Plasma IL-6 was analysed by enzyme linked immusorbent assay (ELISA) using Sanquin Compact Il-6 ELISA kit Cat No: M1916 (Mast Diagnostics, Bootle UK). CRP and VWF were measured using Luminex bead technology in a 2 plex assay in which Magnetic beads (BioRad Watford UK) beads were coupled to US Biologicals Anti VWF monoclonal antbody Cat No: V2700-01E (Stratech, Ely, UK) or Biodesign anti CRP antibody Cat. No: M86842M (AMS Biotechnology, Oxon, UK.). Standards were SCIPAC CRP Cat No; P100-0 (BBI Solutions, Cardiff UK) and US biologicals VWF Cat No: V2651 (Stratech). Biotinylated reagents were Affinity Biologicals Biotinylated Goat Antihuman VWF, Cat No: GAVWF-APBIO (Quadratech Diagnostics, Epsom, UK) and CRP-Biotin**,** biotinylated in house using SCIPAC CRP Cat P100-0 (BBI Solutions). The detection reagent used was R-Phycoerythrin Streptavidin Jackson Immunoresearch Laboratories Cat -16-110-084 (Stratech). All assays were standardised against international standards Cat: IL-6 89/548, CRP Cat 85/606, VWF Cat 09/182 (NIBSC, South Mimms UK ). Levels of sensitivity were 1.92 pg/mL, 0.05 mg/mL and 0.1 IU/mL for IL-6, CRP and VWF respectively.

Additional inflammatory and cardiac biomarkers were available to investigate a subgroup of population A. Inclusion of cardiac biomarkers should support that the propensity scored matched patients suffered similar cardiovascular risk. Neutrophil gelatinase-associated lipocalin (NGAL) was measured on citrated plasma by electrochemiluminescence, using the MESO QuickPlex SQ 120 automate from Mesoscale Discovery Systems (Rockville, Maryland, USA). The lower limit of detection for the assay was 0.015 ng/ml. High sensitive cardiac troponin T (Hs-cTnT) and N-terminal pro brain natriuretic peptide (NT- proBNP) were quantified using electrochemiluminescence supplied by Roche Diagnostics (Indianapolis, USA) For NT-proBNP the lower limit of detection is 0.600 pmol/L and the upper limit of detection is 4130pmol/L. For Hs-cTnT the lower limit of detection for the assay is 0.010 ng/ml.

Auto-antibodies to Anti-Apolipoprotein A-1 (Anti-Apo A1 IgG) and myeloperoxidase (MPO) were analysed using ELISA. Anti-Apo A1 IgG was quantified using arbitrary units (AU). This corresponds to the mean absorbance value of the patient’s serum, minus the control absorbance value (VersaMax, Molecular Devices, California, USA). All biomarker analyses were performed in duplicate. Where extreme inflammatory marker biomarker results were discovered (2SD from mean), clinic letters, urine samples and total white blood cell count from the day of the blood sample were reviewed and data excluded (N=2) where there was documented suspicion of or definitive infection.

Table S1a: Comparative analysis of baseline biochemistry and hematology results split by previous stroke status (Population A)

|  | Previous stroke | |  |
| --- | --- | --- | --- |
|  | No  N = 162 | Yes  N = 157 | p-value |
| Haemoglobin (g/L)^a^ | 118.0 (107.5-128.0) | 120.0 (109.0-132.0) | 0.17 |
| Ferritin (ug/L)^b^ | 116.0 (57.0-252.0) | 149.0 (56.0-316.0) | 0.34 |
| Folate (ug/L)^c^ | 7.9 (6.0-9.6) | 8.0 (5.8-91) | 0.94 |
| Vitamin B12 (ng/L)^d^ | 455.0 (366.0-641.0) | 399.0 (345.0-460.5) | 0.08 |
| Albumin (g/L) | 43 (40-44) | 42 (40-45) | 0.35 |
| Corrected Calcium (mmol/L)^e^ | 2.32 (2.18-2.41) | 2.29 (2.20-2.37) | 0.16 |
| Phosphate (mmol/L)^f^ | 1.14 (0.99-1.29) | 1.12 (0.98-1.32) | 0.10 |
| Parathyroid Hormone (ng/l)^g^ | 96.0 (59.0-154.0) | 67.5 (43.0-133.0) | 0.01* |
| Total Cholesterol (mmol/L)^h^ | 4.00 (3.40-4.70) | 4.00 (3.40-4.90) | 0.80 |
| HDL Cholesterol (mmol/L)^i^ | 1.2 (1.0-1.6) | 1.2 (0.9-1.4) | 0.25 |
| LDL Cholesterol (mmol/L)^j^ | 1.8 (1.6-2.4) | 2.5 (1.8-2.9) | 0.31 |
| Bicarbonate (mmol/L)^k^ | 23.4 (21.8-25.6) | 23.0 (20.4-25.0) | 0.42 |
| Urine Protein Creatinine Ratio (g/mol)^l^ | 35.94 (13.33-129.03) | 44.44 (15.79-128.21) | 0.29 |
| ^Continuous variables expressed as median (interquartile range) and categorical variables presented as number (%). Missing data a=16, b=90, c=233, d=234, e=15, f=18, g=80, h=37, i=37, j=292, k=210, l=3.^ | | | |

Table S1b: Comparative analysis of baseline biochemistry and hematology results split by previous stroke status (Population B)

|  | Previous stroke | |  |
| --- | --- | --- | --- |
|  | No  N = 107 | Yes  N = 127 | p-value |
| Haemoglobin (g/L)^a^ | 119.0 (109.0-128.0) | 121.0 (112.0-132.0) | 0.21 |
| Ferritin (ug/L)^b^ | 116.0 (55.0-263.0) | 125.0 (49.5-258.5) | 0.85 |
| Folate (ug/L)^c^ | 7.6 (6.2-9.3) | 8.4 (6.8-9.4) | 0.29 |
| Vitamin B12 (ng/L)^d^ | 460.5 (352.0-659.0) | 386.0 (30.1.0-461.0) | 0.13 |
| Albumin (g/L) | 43 (41-45) | 42 (40-45) | 0.17 |
| Corrected Calcium (mmol/L)^e^ | 2.23 (2.16-2.36) | 2.25 (2.18-2.34) | 0.72 |
| Phosphate (mmol/L)^f^ | 1.13 (1.00-1.28) | 1.13 (0.98-1.34) | 0.75 |
| Parathyroid Hormone (ng/L)^g^ | 103.5 (65.5-162.0) | 68.5 (44.0-139.0) | 0.01 |
| Total Cholesterol (mmol/L)^h^ | 4.00 (3.40-4.60) | 4.00 (3.50-4.90) | 0.56 |
| HDL Cholesterol (mmol/L)^i^ | 1.2 (1.0-1.5) | 1.2 (0.9-1.4) | 0.57 |
| LDL Cholesterol (mmol/L)^j^ | 1.8 (1.1-2.1) | 2.0 (1.6-2.5) | 0.50 |
| Bicarbonate (mmol/L)^k^ | 23.7 (20.8-27.5) | 22.8 (20.4-25.0) | 0.42 |
| Urine Protein Creatinine Ratio (g/mol)^l^ | 33.33 (14.81-97.09) | 40.00 (15.38-120.00) | 0.39 |
| ^Continuous variables expressed as median (interquartile range) and categorical variables presented as number (%). Missing data a=12, b=85, c=180, d=181, e=12, f=14, g=32, h=23, i=15, j=217, k=139, l=2.^ | | | |

Table S2: Univariate associations with all-cause mortality, ESRD and future NFCVE.

|  | All-cause Mortality | | | | | | Reaching ESRD | | | | | | Future NFCVE | | | | | |
| --- | --- | --- | --- | --- | --- | --- | --- | --- | --- | --- | --- | --- | --- | --- | --- | --- | --- | --- |
|  | Total Population N=319 | | No previous stroke N=162 | | Previous Stroke N=157 | | Total Population N=319 | | No previous stroke N=162 | | Previous Stroke N=157 | | Total Population N=319 | | No previous stroke N=162 | | Previous Stroke N=157 | |
| Number of events: | 175 (54.9%) | | 72 (44.4%) | | 103 (65.6%) | | 117 (36.7%) | | 56 (34.6%) | | 61 (38.9%) | | 55 (17.2%) | | 13 (8%) | | 42 (26.9%) | |
| Variable | HR (95%CI) | p-Value | HR (95%CI) | p-Value | HR (95%CI) | p-Value | HR (95%CI) | p-Value | HR (95%CI) | p-Value | HR (95%CI) | p-Value | HR (95%CI) | p-Value | HR (95%CI) | p-Value | HR (95%CI) | p-Value |
| Male Sex | 1.547 (1.099-2.178) | 0.012 | 1.370 (0.816-2.302) | 0.234 | 1.695 (1.075-2.875) | 0.023 | 2.977 (1.797-4.931) | 0.000 | 2.466 (1.241-4.898) | 0.010 | 3.580 (1.695-7.561) | 0.001 | 1.404 (0.787-2.503) | 0.251 | 1.031 (0.334-3.183) | 0.957 | 1.661 (0.840-3.283) | 0.144 |
| Age (per year) | 1.044 (1.025-1.064) | 0.000 | 1.062 (1.032-1.092) | 0.000 | 1.032 (1.008-1.056) | 0.007 | 0.987 (0.959-1.005) | 0.146 | 0.999 (0.967-1.032) | 0.932 | 0.977 (0.954-1.001) | 0.057 | 1.036 (1.002-1.071) | 0.035 | 1.106 (1.032-1.185) | 0.004 | 1.018 (0.985-1.053) | 0.292 |
| Living alone | 0.740 (0.506-1.082) | 0.121 | 2.554 (1.421-4.588) | 0.002 | 0.953 (0.563-1.612) | 0.858 | 0.896 (0.527-1.523) | 0.896 | 0.964 (0.406-2.287) | 0.933 | 1.023 (0.516-2.030) | 1.023 | 1.127 (0.621-2.048) | 0.694 | 0.410 (0.781-7.435) | 0.126 | 0.796 (0.389-1.629) | 0.533 |
| Smoking history | 1.660 (1.165-2.364) | 0.005 | 1.462 (0.872-2.452) | 0.150 | 1.890 (1.147-3.115) | 0.013 | 0.981 (0.653-1.473) | 0.925 | 0.961 (0.546-1.693) | 0.961 | 0.744 (0.386-1.433) | 0.377 | 0.825 (0.477-1.426) | 0.491 | 0.677 (0.226-2.023) | 0.484 | 0.893 (0.467-1.706) | 0.732 |
| Diabetes | 1.107 (0.821-1.492) | 0.506 | 1.238 (0.779-1.968) | 0.367 | 0.984 (0.665-1.457) | 0.936 | 1.228 (0.853-1.738) | 0.270 | 1.405 (0.815-2.420) | 0.221 | 0.855 (0.511-1.429) | 0.549 | 1.403 (0.816-2.412) | 0.221 | 0.676 (0.206-2.219) | 0.519 | 1.739 (0.919-3.294) | 0.089 |
| Previous myocardial infarction | 1.467 (.068-2.015) | 0.018 | 2.125 (1.318-3.428) | 0.002 | 1.058 (0.687-1.630) | 0.799 | 0.792 (0.505-1.241) | 0.308 | 1.027 (0.540-1.952) | 0.935 | 0.645 (0.342-1.215) | 0.175 | 2.186 (1.276-3.745) | 0.004 | 3.557 (1.183-10.696) | 0.024 | 1.598 (0.85-2.999) | 0.144 |
| Heart failure | 1.470 (1.087-1.989) | 0.012 | 1.656 (1.037-2.643) | 0.035 | 1.058 (0.687-1.630) | 0.799 | 1.069 (0.727-1.572) | 0.736 | 1.062 (0.621-1.818) | 0.825 | 1.103 (0.629-1.935) | 0.733 | 1.135 (0.654-1.971) | 0.653 | 1.980 (0.663-5.914) | 0.221 | 0.836 (0.431-1.623) | 0.597 |
| Peripheral Vascular Disease | 1.529 (1.125-2.079) | 0.007 | 1.733 (1.084-2.770) | 0.022 | 1.386 (0.22-2.082) | 0.116 | 0.891 (0.608-1.306) | 0.554 | 1.119 (0.653-1.917) | 0.684 | 0.674 (0.386-1.176) | 0.165 | 1.847 (1.066-3.201) | 0.029 | 1.910 (0.639-5.707) | 0.247 | 1.799 (0.953-3.395) | 0.070 |
| eGFR (per 5mL/min/1.73m2 decrease) | 1.385 (1.264-1.517) | 0.000 | 1.515 (1.291-1.777) | 0.000 | 1.355 (1.240-1.519) | 0.000 | 2.613 (2.176-3.137) | 0.000 | 2.411 (1.897-3.064) | 0.000 | 2.411 (1.897-3.064) | 0.000 | 1.115 (0.92-1.26 | 0.092 | 1.004 (0.788-1.278) | 0.976 | 1.216 (1.039-1.423) | 0.015 |
| uPCR (per 10mg/mmol) | 1.017 (1.008-1.026) | 0.000 | 1.014 (1.001-1.027) | 0.038 | 1.023 (1.009-1.037) | 0.001 | 1.0035 (1.027-1.043) | 0.000 | 1.032 (1.020-1.043) | 0.000 | 1.027 (1.013-1.041) | 0.000 | 1.019 (1.00-1.039) | 0.054 | 0.968 (0.890-1.054) | 0.459 | 1.037 (1.014-1.061) | 0.002 |
| Albumin (per 1g/mL) | 0.908 (0.871-0.948) | 0.000 | 0.911 (0.850-0.976) | 0.008 | 0.914 (0.865-0.965) | 0.001 | 0..927 (0.880-0.977) | 0.005 | 0.891 (0.834-0.953) | 0.001 | 0.977 (0.907-1.051) | 0.529 | 0.857 (0.795-0.924) | 0.000 | 0.796 (0.670-0.946) | 0.010 | 0.893 (0.820-0.974) | 0.010 |
| ILl6 (per SD) | 1.529 (1.275-1.832) | 0.000 | 1.607 (1.214-2.128) | 0.001 | 1.461 (1.153-1.850) | 0.002 | 1.095 (0.880-1.363) | 0.415 | 1.037 (0.755-1.423) | 0.924 | 1.146 (0.848-1.548) | 0.376 | 1.263 (0.815-1.745) | 0.156 | 1.683 (0.877-3.232) | 0.118 | 1.103 (0.757-1.606) | 0.610 |
| VWF (per SD) | 1.364 (1.149-1.619( | 0.000 | 1.568 (1.212-2.027) | 0.001 | 1.176 (0.928-1.491) | 0.179 | 1.010 (0.822-1.242) | 0.922 | 1.269 (0.951-1.694) | 0.106 | 0.634-1.147) | 0.293 | 1.254 (0.916-1.717) | 0.157 | 1.768 (0.962-3.249) | 0.067 | 0.957 (0.643-1.424) | 0.828 |
| CRP (per SD) | 1.388 (1.183-1.628) | 0.000 | 1.373 (1.079-1.747) | 0.010 | 1.390 (1.123-1.721) | 0.003 | 1.004 (0.823-1.224) | 0.971 | 0.910 (0.684-1.211) | 0.519 | 1.087 (0.822-1.438) | 0.557 | 1.044 (0.784-1.390) | 0.769 | 0.956 (0.528-1.732) | 0.882 | 1.063 (0.763-1.480) | 0.720 |
| Missing values: UPCR =3 (2 in stroke group). | | | | | | | | | | | | | | | | | | |
